# “My Core Is Cracked”—Bullying in Higher Education as a Traumatic Process

**DOI:** 10.3390/ijerph21111462

**Published:** 2024-11-01

**Authors:** Margaret Hodgins, Declan Fahie, Sarah MacCurtain, Rhona Kane, Patricia Mannix McNamara

**Affiliations:** 1 Health Promotion Research Centre, University of Galway, H91TK33 Galway, Ireland; 2 School of Education, University College Dublin, DO4C7X2 Dublin, Ireland; declan.fahie@ucd.ie; 3 School of Education, University of Limerick, V94T9PX Limerick, Ireland; sarah.maccurtain@ul.ie (S.M.); patricia.m.mcnamara@ul.ie (P.M.M.)

**Keywords:** workplace bullying, higher education, traumatic process

## Abstract

Sectoral and institutional context[s] are critical to understanding how workplace toxicity manifests and how it might best be addressed. The education sector, specifically higher education, is the focus of this study, drawing on qualitative data collected from Irish Higher Education Institutions [HEIs]. Underpinned by a multi-faceted conceptualisation of bullying, the study explores how it is experienced by university staff and how institutional or contextual factors impinge on that experience. The study employed a qualitative interpretive methodology involving one-to-one semi-structured interviews with self-selecting participants. Persons who currently work or have recently worked in higher education institutions were recruited into the study. A generic thematic approach resulted in five intersecting themes, converging on one overarching organising construct, i.e., bullying in higher education as a traumatic process. The data displayed relationality, institutionalisation and unethicality, which are underlying features of a multi-faceted conceptualisation of bullying. It was concluded that the processes and procedures in place to address bullying and provide redress do not appear to be sufficiently nuanced to accommodate the complex behaviours and power plays involved in bullying in Higher Education, assuming a rationality stripped of power dynamics, which risks aggravating the damage already inflicted by bullying. The findings suggest that the institutional response, or lack thereof, can sometimes be more traumatising than the bullying itself. Individual cases often reflect a wider organisational culture that tolerates bullying and victimisation. Institutional responses will need to tackle the entire HEI ecosystem, requiring and a more nuanced understanding of the power dynamics and organisational context.

## 1. Introduction

Workplace bullying [WB] is a complex phenomenon. Despite the many definitions and analogous terms in the literature, there is a general consensus that workplace bullying is an especially detrimental form of aggressive or abusive behaviour, systematic, persistent and targeted [1,2,3]. The behaviours employed are wide-ranging; for example, interpersonal and emotionally abusive behaviours include verbal cruelty, predation and micro-political “game playing” for personal gain [4,5], while work-related behaviours refer to the more subtle enactment of punitive and unfair management practices, such as exclusion from key processes or assigning unreasonable duties [4,6]. It is the processual, targeted and deliberate nature of workplace bullying that distinguishes it from day-to-day aggravations or conflicts, resulting in compromised health and well-being [7], sickness absenteeism [8] and sickness presenteeism [9]. Despite this, workplaces struggle to effectively prevent or ameliorate it.

The impact of workplace bullying on health and well-being is unequivocal and consistent; meta-analyses of studies employing indicators of both physical and mental ill-health reveal a robust relationship between the experience of being bullied and compromised health and well-being [7]. Although there are relatively few qualitative studies specifically addressing the impact of workplace bullying on health, negative impacts inevitably emerge in qualitative studies exploring the experience of being bullied; these include, for example, lowered self-confidence, feelings of self-contempt, guilt, isolation, vulnerability [10,11,12,13], and a challenging of targets’ basic assumptions about themselves and others [14], including identity disruption [15]. Much of the focus of health impact studies relates to exposure to bullying behaviours, with less attention paid to the contribution of the organisational response.

Although workplace bullying has a universal presence across all workplaces globally [3], place and space matter. Nielsen and Einarsen, [1,2] report an average prevalence of workplace bullying of 14.7%, while cautioning against comparisons without due consideration of the methodological moderators of location, instrument and sampling strategy. There is considerable variation by occupational sector, with the health and social care, defence and educational sectors displaying higher than average levels of exposure [6,16,17,18]. Both sectoral and institutional context[s], therefore, are critical to understanding how workplace toxicity manifests and how it might best be addressed. The education sector, specifically higher education, is the focus of this study. The prevalence of workplace bullying in higher education is at least commensurate with the levels of exposure generally, and is likely higher [19], bearing in mind the difficulties inherent to comparison. Specific contextual factors include the hierarchical nature of academic organisations [20], a culture of critique, entitlement and low accountability [21], the neoliberal agenda that drives managerialist imperatives, hyper-competition and the silencing of bullying from prominent researchers or those with significant influence over career trajectories [20,22,23,24]. Critically, the nebulous and nuanced nature of these contextual factors can evade quantitative measurement and, we tender, will be better captured by qualitative methodologies.

This article draws on qualitative data collected across the Irish HE sector, which has received limited research attention, despite the ingress of neoliberal policies in recent years [25]. Underpinned by D’Cruz’s multi-facetted conceptualisation of bullying [3], this study explores how bullying and the response to it is experienced by university staff and how institutional or contextual factors impinge on those experiences.

## 2. Workplace Bullying in Higher Education

Workplace bullying is a notoriously difficult and contested construct. The many definitions offered [26,27] and related constructs reflect the different emphases, rather than substantive conceptual distinctiveness. Bullying is clearly complex, compound and multi-faceted [3,27,28,29], although the most frequently employed definitions [29,30,31] are consistent in referring to a process or pattern of aggressive, abusive or harassing behaviours that are systematic and targeted, from which targets feel unable to escape and that are damaging to their health, well-being, self-respect and identity.

Bullying presents as aggressive or abusive behaviour systematically enacted by one or more perpetrators, towards a target, over a period of time. Since bullying includes subtle tactics and abuses of power, in the context of particularised social relationships, filtered through the lens of subjective interpretation on the part of both target and investigator, it can be difficult to identify and prove [32].

It is challenging to arrive at a general estimate of the prevalence of workplace bullying, given the variations in approaches to definition and measurement. Nielsen et al. have demonstrated the moderating effect of method, with self-labelling studies (a single item question asking whether the respondent has been bullied or not within a given time period, with some studies providing a definition and others not) yielding average estimates of either 10.6% or 19.8% with or without a definition, respectively, and 14.7% using a behavioural checklist (respondents complete an inventory that lists various types of unwanted negative behaviours with response options: never, occasionally, monthly, weekly, daily, etc.) [33,34]. Each method has both advantages and limitations, but critically, if comparisons across time or sector are sought, measurement method needs to be taken into account [34]. A small number of studies provide comparative figures for different sectors or workplaces using the same instrument, and these indicate that HEIs cannot be complacent when it comes to workplace bullying. Using the behavioural checklist approach, 19% and 16.6% of the staff of the Italian University and Norwegian University sectors, respectively, were deemed to have experienced bullying [35,36], higher rates than comparator organisations. Keashly, reviewing the literature from the US, offers an overall estimate of bullying rates in higher education of ca. 25%, 5–10% higher than rates in the general population [19,37].

The higher prevalence in universities can at least in part be understood in terms of well-established institutional factors that predispose certain organizations to bullying and coalesce in HEIs. Large organizations, hierarchical organizations [38] and public sector organizations [39] are vulnerable to a higher prevalence of bullying. Crucially, all three features are present in HEIs. The hierarchical nature of HEIs reaches beyond the institution itself as academics also work within networks of disciplines outside of their organisation, which can create ‘split loyalties and responsibilities’ [40] and exacerbate power differentials. Male-dominated organisations are also subject to higher rates of bullying [41]; again, this is a well-established feature of HEIs as universities are predominantly run and managed by men [42,43,44]. Early work on bullying observed that bullying is more likely in “total” organizations, where ‘dominance and power imbalances are strongly emphasized’ [38] (p. 1219) and where there is a strong emphasis on rank, authority, and conformity [45], or which are competitive and politicized. These conditions are usually associated with institutions such as the military or prisons, but a ubiquitous neoliberal ideology which inscribes corporatisation and managerialism throughout the higher education sector foregrounds these conditions within higher education nationally and internationally.

The negative impact of bullying on worker health is well established. Meta-analyses of both cross-sectional and longitudinal studies demonstrate a robust relationship between being bullied and experiencing compromised physical and mental health [2,7]. Cross-sectional, multivariate and longitudinal studies have demonstrated that bullying predicts reduced or poorer health, while poor health also raises the risk of exposure to bullying. Qualitative studies reinforce the damage of bullying and demonstrate its nuanced impact, exposing how bullying is a social relational problem in which relationships are both causes and casualties, whereby targets lose trust in their work group or the organisation, become disillusioned, and experience shame and/or compromised professional identities [5,15,46,47]. Qualitative studies reveal that bullying impacts self-worth, and can engender feelings of self-contempt, guilt, isolation and vulnerability [11,13,48]. Findings pertaining specifically to HEIs have found that incivility and bullying can lead to health problems including anxiety, stress, depression, and alcohol abuse [49]. The detrimental impact of bullying is not restricted to the target alone and can also have significant consequences for other staff and the student body [50,51].

Finally, despite evidence that bullying has a substantial negative impact, both at the aforementioned level of individual health [2,10,12,52] and organizational productivity and costs [53,54,55], organisations typically fail to prevent or ameliorate it. Barratt-Pugh and Krestelica [50] refer to bullying in HEIs as culturally resilient, despite extensive policy regimes; this is due to hierarchical and bureaucratic structures embedding power inequalities that are further intensified by the move towards a more competitive, individualistic and managerialist model. They argue that, while anti-bullying policies are important, they only represent the first stage in changing HEI cultures and are futile without ‘authentic’ management intent. The evidence that workplaces are ineffectual in protecting workers from bullying is drawn from studies on turnover intention and quit rates, studies on self-reported actions taken [or not taken], qualitative accounts of targets’ experiences of organizational responses, and studies on HR team’ perspectives on organizational response [56,57,58]. Workers have a significant need and legitimate expectation for fairness and reasonable protection in their workplaces. When it fails to materialize, and worse, when the perpetrator of psychological abuse is not reprimanded or sanctioned, this has the potential to exacerbate already compromised health.

In the context of the higher prevalence of bullying in universities and evidence of a poor organizational response, this study explores how bullying and the response to it are experienced by university staff and how institutional or contextual factors impinge on those experiences.

## 3. Methods

This study employed a qualitative interpretive methodology involving one-to-one semi-structured interviews with self-selecting participants. The interview schedule had three sections; the first asked participants to describe their experience of bullying while working at a HEI, the second explored perceived impact on health and, finally, a third section addressed perceptions about bullying and the HEI work environment. The interviewer was an experienced communicator with a background in health care and training in research methods.

Persons who currently work or have recently worked [thus allowing for persons who have exited their job due to bullying] in higher education institutions, such as academic staff, researchers and professional staff, including HRPs and senior management, were recruited into the study. The sample was self-selecting for HEI staff who have experienced workplace bullying while working at an Irish university or third-level institution. The invitation was phrased in this way to allow the respondents to be either targets, witnesses or alleged perpetrators.

Recruiting participants in studies of workplace bullying is challenging [59]. They are a vulnerable population insofar as they are highly likely to be experiencing, or have experienced, compromised health; thus, a high level of sensitivity and compassion was deemed critical during the interview. In order to minimise the risk of exposure to further bullying, which could occur if recruiting via individual institutions, participants were recruited through social media. An invitation was issued via Twitter (between May and July 2023) with a link to an MS form, which provided a participant information sheet and participant consent form. Consenting participants were forwarded to the email address of the principal investigator, who subsequently set up an interview with a research interviewer. Once the interview had taken place, a pseudonym was applied and used for the subsequent discussion, analysis and presentation of results (the study was approved by the University of Galway Research Ethics Committee.).

As the study did not align precisely with the foundational qualitative approaches of phenomenology, ethnography or grounded theory [60], we identify it as a generic qualitative approach [60] underpinned by Caelli et al.’s criteria [61]. Data analysis followed Braun and Clarke’s six-step process [62] and we used NVIVO software (NIVIVO 12) to store and sort the data. Transcripts were uploaded to NVIVO, read and re-read by a minimum of two team members/researchers. Each transcript was summarized, drawing on field notes as appropriate to achieve data familiarisation. The data were coded and the codes were re-constructed within and across transcripts into meaning units or themes. In this way, the generation of themes was data driven.

Persons who had experienced workplace bullying while working at an Irish university or third-level institution were invited to contact the principal investigator as outlined above. Ten digitally recorded interviews were completed. Eight of the ten participants were prepared to provide brief demographic details. Three had worked between 3 and 5 years in the sector, three 11–20 years and two over 21 years. Of the eight, six were academic staff and two were professional staff. Seven of the eight participants who provided demographic information identified as female. The ten in-depth interviews which inform this study revealed rich insights into the participants lived experiences. Their generosity, thoughtfulness and sincerity mitigated any concerns with respect to the size of the sample, which can often be a factor when researching a sensitive topic like toxic interpersonal relationships at work (see, for example, [59]). The feminised profile of the sample is consistent with higher rates of exposure to bullying for female staff in higher education [37], likely a function of the over-representation of women in subordinate positions and the under-representation of women in powerful positions in higher education institutions internationally [20], and particularly in Ireland [42].

**Figure 1:**
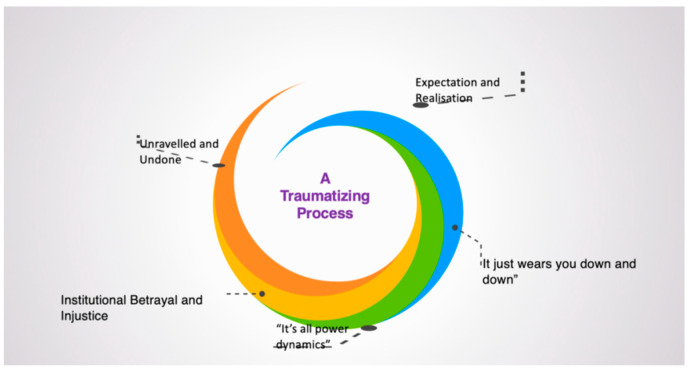
Five themes and central organising construct.

**Figure 2:**
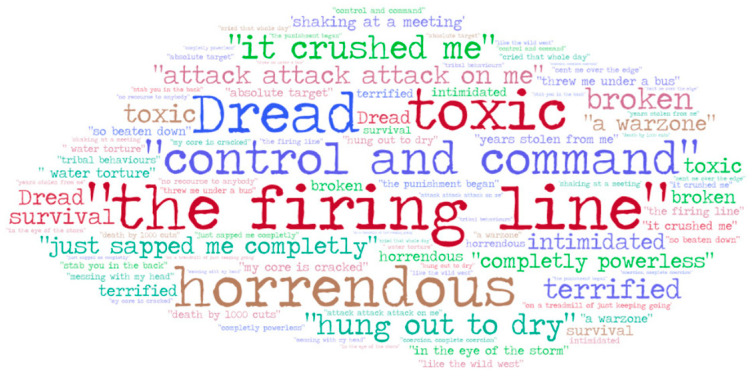
Words, phrases and metaphors used in participant interviews.

Interview transcripts generated 288 pages of text. The interview times ranged from 45 min to 1 h 54 min, with an average duration of one hour. The approach to analysis was a generic thematic approach [61], resulting in five intersecting themes, converging on one central organising construct [63]: bullying in higher education as a traumatic process (see Figure 1). Aphoristically, we chose to simply present words used by participants in the course of their interviews to illustrate the traumatic nature of their experience (see Figure 2). The contributing themes are then described in detail.

## 4. Results

This study explores the process of WB in HEIs. In particular, it focuses on the negative impact of toxic behaviours on employee health. In this context, the following quote from Dara illustrates how bullying was not expected, or what people thought it might be. Participants did not quickly jump to the conclusion they were being bullied. On the contrary, they sought other interpretations for the behaviour of the would-be perpetrator. Typically, there was a lead-in period, characterised by self-blame, uncertainty or a re-assessment of beliefs about bullying.

*“I always think when you see the stuff around campus around ‘Bin Bullying’ and all this stuff, that if you came across bullying, it’d be really obvious and you’d be able to say ‘She did this or he did that. You clearly crossed the line’. My experience of it was completely different. It was subtle. It was devious. It was vicious and it had desperate consequences on people’s lives”.*

Miscommunications, someone having a bad day, and someone not being a particularly competent manager were initial interpretations of bullying. In these instances, the targets focused initially on their own performance, believing that they could be the problem. Anna, for example believed the following: ‘*If I just do my job really well, then he’s gonna stop treating me like this*’. Dara similarly engaged in self-blame: *‘Well, at the start I certainly felt like it was something wrong with me. I think probably a lot of people do that when you when these things happen. It’s like I’m not managing this well and that was that was my motivation for going to speak to the therapist was. I need to figure out a way for me to cope. And to be resilient. Myself, in other words, my issue, my problem…’.*

Grainne also commented on her internal disbelief that undermined her own rational judgement.

*“A couple of months after I started I noticed that there were back-handed compliments being given by my direct supervisor… Anytime I’d ask a question, my supervisor would start to roll her eyes and invalidate anything that I’d ask, or anything that I’d say. But not outwardly. It wasn’t very obvious to everybody, but it was kind of a pattern of her dismissing anything that I said’*.

Eva reported being bullied over a 10-year period, before she recognised it. Only when prompted by colleagues did she recognise that the demands being placed on her were unachievable, personalised and ‘just bonkers’.

In cases where their job was a promotion, e.g., transitioning from being a post-doc or to an innovative project, participants felt particularly cheated by the experience of being bullied. Chris’s expectations were high: ‘*I thought I can’t wait to go working for this person because she was so dynamic I thought oh I’m going to be so happy here and I it was always my dream to work in higher education as well…*’. Later, however, Chris recounted the following: ‘*… I was so beaten down there … it just sapped you completely*’. Ita similarly described her job as a ‘dream’ job, but was forced to leave due to bullying, a situation that was grievously distressing for her.

### 4.1. It’s All Power Dynamics

Participants’ experiences of bullying varied considerably. Several situations involved perpetrators who were senior academics; however, there were also situations recounted that were better characterised as upward bullying or mobbing by a group of peers. Both academic departments, labs and specialised units provided contexts for bullying. However, regardless of the context and situation, as Fran stated—‘*it’s all power dynamics*’. Participants consistently identified issues of power and control as central to their experience of being bullied, repeatedly using words such as ‘belittling’, ‘undermining’, ‘excluding’, ‘undercutting’ and ‘blocking’. Both position[al] power and social or emotional power were evident.

What is striking is that once targets start to recognise bullying, they are in no doubt that the perpetration of bullying is an exercise of power and that they are being targeted in order to reduce or circumscribe their power, thus rendering them impotent. Bullying was interpreted primarily as a pernicious exercise of power as opposed to an escalating aggression or conflict. Holly sums this up by saying:

*“… one thing that I’ve really learned (is) that the difference between interpersonal conflict and bullying for me is really about (a) power imbalance and making the other person feel powerless. And that’s very different to just a difference of opinion over things”.*

For the participants here, power was exercised in an abusive manner, knowingly and intentionally. The analogies drawn by participants are particularly telling with respect to their recognition of power dynamics. Parallels with playground bullying, the stock market floor, domestic violence and coercive control are drawn, for example, while Fran likens the environment to a primitive ‘tribal society’, where alpha males give a show of power, signalling their position in the hierarchy and their intention to control the group. Eva reflects similarly: “… *he was in a position of power the whole time and knew it. …even so much so that he said about what great friends we were, he said, but he goes, but I hold the power”.* Position[al] power was exercised in the form of line managers bullying targets through exclusionary tactics, the micromanaging of work, questioning targets’ expertise in a public forum, redirecting intending PhD students away from the target, withholding information from the target about networks or meetings and speaking ‘for’ the target. Fran recounted the following:

*“… just like everything they could do to block you, you know. So, whether it’s access to a room that you need or it’s access to materials or equipment. It is, you know, ignoring the emails that you’re sending, not including you, in important meetings where things are being discussed”.*

Changing power dynamics in the HEI often result from professional envy on the part of the perpetrator when, for example, the target develops an independent research profile. This results in what may be seen as a challenge to their superior’s dominance and professional status. Anna comments were as follows: *“… we were quite research-aligned and so… me coming in to the department was a bit of a threat to him and he really wanted me to more act like a senior postdoc in his group rather than pursuing my own research interests”.*

A particularly sinister exercise of power, gaslighting, was identified by participants. In Eva’s case, the perpetrator kept offering her additional work opportunities; yet, when she undertook the work, she was belittled and undermined. He told her how important she was, yet also led her to question herself and attempted to manipulate her into believing she was, in fact, the cause of his bullying. Detailing her interactions with him, she recalls the point when she realised this: “*… whoa. There it is. There it is. You know, he’s sorry. But he’s not really sorry because I drove him to it*”.

Targets experienced powerlessness, recognising that the power differential was embedded within the organisational structure, and that the perpetrator had the implicit sanction of the institution behind them. This was particularly salient when the target was a precarious worker. Eva explains:

*“… I really needed the job, but he was in a position of power… so when things began to happen, I couldn’t address it directly because I needed to work and he was in in a position to give me further work and also to ensure that I was going to progress in my career…I was holding a meeting because I was in charge of something. And [perpetrator] kept interrupting me. And this is something he does all the time and then correcting me and interrupting me and then said you don’t know how to run a meeting, do you? And as we were in front of everyone and … I took it. I just took it…because I had no power”.*

Ita too, felt powerless in the face of humiliating treatment: “… *those men, like basically, here we’ll throw tomatoes at her and the rest of us will just sit back this is an hour, two hours entertainment for us*”.

The exercise of social and emotional power was evident in Breda’s account of a vicious email campaign by a group of peers, Holly’s account of upward bullying from a former colleague and both Fran and Ita’s accounts of a phalanx of male colleagues resisting collaboration/cooperation on institutionally sanctioned changes to their way of working. Threat was perceived to be behind these situations also. Ita, in retrospect, acknowledges the threat she posed: “*I probably scared the shit out of them… So I didn’t realize I was creating fear for them. I didn’t realize that… but I know now*”.

Participants recognised that power was exercised both overtly and covertly. For example, the power to actually define a situation was noted:

*“I think during the period where it was like, oh, if I ever wanted to get promoted again, this guy’s got to be part of it. Well then why bother? That was frustrating as well to feel like no matter how good I was that this person with power could always just say, well, I think that what Anna’s doing is stupid and everyone would be like, oh yeah, I guess it is then”.*

One aspect of the abuse of power, unique to this setting, was the use of students as a proxy, an experience which participants found particularly disturbing. Poaching graduate students, discrediting the target as a potential supervisor, sabotaging assessment practices and interfering with recruitment practices were highlighted in interviews and described by participants as deeply upsetting and unprofessional.

While targets identified and described feelings of powerlessness, this is not to say that they did not exercise power. Most participants sought intervention from the institution in some form, and several made formal complaints, which we explore in later themes. However, the process of bullying did include a lived experience of powerlessness at least at some point in the process, with the recognition of the risk that addressing it posed to health and well-being.

### 4.2. It Just Wears You Down and Down and Down

While bullying experiences ranged in nature and duration, a pattern of sustained, constant exposure to bullying behaviours was evident. These negative experiences were both sustained and constant, and seriously impacted participant health. Participants experienced bullying as unremitting and unrelenting. Participants argued that while the behaviours might not, at first glance, seem toxic, their corrosive impact is compounded by daily unabated exposure. Breda described her situation as follows:

*“… You could have the strongest bit of rock to start with. But if you keep chipping away at it… it will just wear down over time… If somebody else told me this was happening, they might’ve said ‘oh, go away with that’… And people did say that to me… I think people meant well, but I don’t think they fully understand the impact. Because they weren’t having this at least 3 days a week… some weeks it was more, and over a long period of time… it just wears you down and down and down…”.*

The impact of the incessant “wearing down” of targets is significant. Even in the absence of a specific interaction with the perpetrator, the anticipation of a potential interaction is injurious, as Wendy pointed out: “… *you’re waiting for the onslaught*”. For Anna, the sustained and consistent undermining of all aspects of her work is what drove her to seek redress. Ita and Wendy spoke of how the bullying profoundly undermined their self-confidence. In Ita’s case, this led her to leave the institution: *“… I thought, you know what, I’ve had enough”.*

Holly, a victim of upward bullying, used the term *‘death by a thousand cuts’*, drawing attention to the stress on a day-to-day basis of trying to continue working and ‘pretend’ it was not going on. She also recounts her own hypervigilance and her concern about others making complaints about her and, ultimately, about the possibility of losing her job. She was overwhelmed by the experience, debilitated and at the point of breakdown.

Across all interviewees, health was seriously impacted, with anxiety featuring frequently in accounts of daily exposure to bullying. Anxiety, interviewees argued, could lead to either mental or physical illness or, indeed, both. For those interviewed for the study, these poor health impacts were a direct result of the quotidian nature of their experience. Fran, for example, described the impact of two years of persistent bullying as profound: “*Chronic insomnia, extreme anxiety, you know, constant like, I could say, since I started working in that place there isn’t a day that hasn’t gone by where I haven’t felt sick in my stomach. Profound*”. Breda recounts suicidal thoughts brought on by her experience, saying that at one point, she felt so hopeless that her institution was “*only five minutes away from having blood on its hands*”. Chris still has stomach pains just thinking about experiences that were 2 years prior, while Grainne experienced on-going stomach problems due to anxiety brought about by constantly worrying and double-checking everything she did. In this way, anxiety appears to operate as a transitional belt to physical symptoms in addition to mental distress. For Wendy, the experience was potently visceral:

*“… you were just constantly in the eye of the storm. I got to the point where I couldn’t hold down food when I was on campus. I just couldn’t eat. So physically crossing the barrier of campus that was it. I wouldn’t be able to eat again. I’d literally would vomit. And then, if I left campus, if I walked across the road to have lunch across the road, I could eat…and it was it was absolutely physical, I remember driving into work and sitting in the car park and thinking I cannot get out of my car…and driving home again, I mean it was constant…”.*

### 4.3. Institutional Betrayal and Injustice

All ten participants attempted to resolve the bullying situation, either informally through a line manager, trade union or HR, or through engaging in a formal grievance process. For each participant, their experiences of organisational response were negative, with reactions ranging from frustration to blind fury. The difficulties with the organisational response included a demonstrable poor understanding of bullying, the ignoring of previous issues with the perpetrator and a seeming reluctance to apply sanctions to the perpetrator, even where the grievance was upheld in favour of the target. Such responses, participants argued, served to retraumatise the targets.

Some participants were surprised at proposed resolutions to bullying, having expected a greater understanding from managers and/or HR. Examples include suggesting that, since the target had successfully been promoted during the time she raised the issue, that it was no longer an “issue” [even though she remained in the hostile environment]; or after a graduate returned to work following sick leave due to bullying, the perpetrator was nominated by HR as a suitable supervisor of their return-to-work process. Participants recounted HR telling the perpetrator not to communicate with the target, ignoring the fact that this was neither possible nor effective, or being sent from HR to unit or line managers who sent them back again to HR, with no one taking responsibility for action.

Essentially, participants interpreted poor responses as fundamentally disrespectful and as exacerbating their distress. Breda called the organisational response as the ‘*biggest disaster ever*’, describing being met by HR in an inappropriate space, with someone who did not have any details and then gave her incorrect advice. Furthermore, participants recounted stressful delays in the process, which they viewed as failure on the part of the HEIs to adhere to their own policies and procedures. Wendy only managed to secure a meeting with management about her complaint when she was at the point of taking maternity leave, despite having requested a meeting several months previously:

*“They just didn’t respond to anything again. It was all delay, delay, delay…they just delayed everything. So, I put it in writing- my complaint. They didn’t respond, and even that, I felt was bullying because they knew I was trying to get it addressed quickly, because I was pregnant and they I dragged it up onto the day I was going on maternity leave. Even that process, I felt, was an abuse of me…I mean that because HR had a timescale, but they never stuck to. What should have taken 30 days, took almost 5 months, 6 months, when I didn’t have 5 or 6 months…My entire pregnancy was overshadowed by this, which I resent to this day”.*

Ignorance of how such ineffective responses impact individuals was noted by Breda, who opined that “… *organization-wise, I think maybe people don’t get it fully*”. Crucially, the mismatch between the official timeframes detailed in intuitional polices and the reality of long delays and silences was viewed as a concrete example of the poor understanding of WB by HR departments. Holly’s details her own experience:

*“I was told the process would take 2 to 6 months. And I kept telling myself, look, I just have to get over a few months here and it could be done and dusted. But it took 18. If I’d have known at the start it’s going to take 18. I’d have gone to the doctor sooner. I’d have reached out more. I’d have been more proactive maybe about support, realizing after 3 or 4 months I’m going to have another year of this so I need to take care of myself. instead of thinking after 3 or 4 months the thing is nearly over when it absolutely wasn’t. … you were on this treadmill of telling yourself ‘I just have to get through another day, another week and then, but like I had little did I know at the time I still had over a year to go…”.*

Where participants referred to being offered mediation, none believed or expected mediation could work. Indeed, participants were wary of the mediation process, questioning its suitability for serious cases of WB. Being told that you should enter mediation with someone who is clearly bullying you is, in Anna’s words, “*kind of ridiculous*”. Eva too, emerging from a situation of protracted coercive control, also resisted mediation, knowing at that stage that the perpetrator would manage to out-talk her. Fran described an attempt at mediation which failed utterly, again revealing a lack of understanding on the part of HR with respect to power dynamics:

*“She tried to do this kind of mediation, and his behavior was utterly shocking. I couldn’t believe what I was witnessing. He’d sit there like this, you know, with the legs open… between myself and her… she trying to conduct a mediation and she’d be almost crying… she wasn’t able to manage it at all”.*

Ita’s experience was particularly upsetting, being blamed for causing trouble in an institution that claimed it had never had a confirmed case of bullying in the past:

*“The line manager and the head of HR… It was attack, attack, attack on me. I sat through that meeting for, I’m sure, 3 hours… it was very evident from HR that they were defending the line manager and his decisions… [saying] nobody had complained ever before and that you know here I was a troublemaker coming in and causing trouble in the organization…”.*

Several participants noted that the perpetrator had previously been accused of inappropriate behaviour[s]. It was a source of incredulity that the organisation could not, or would not, be alert to these patterns of allegations and, as a consequence, allowed staff to be exposed in this way. Chris maintained that 20 people had left after working with the perpetrator in that HEI. The failure to act proactively was maddening and hurtful for staff who could only interpret this as them being just one more unimportant or dispensable victim. Fran had a particularly frustrating experience:

*“It was well known! There was a long-standing history of problems with him… and when I went in there… I remember [Head of Dept] coming down to the canteen. She sat down one day, and she said ‘Oh, how’s it going with [perpetrator]?’ I said ‘fine’, and I think there was a kind of a weird, curiosity as to how the hell I was managing to work with him, you know… What I found out was that there was absolutely horrendous kind of complaints against him… colleagues had lodged numerous complaints, that there was a whole file cabinet full of it…”.*

Relatedly, there were instances where the perpetrator was promoted or had a term of office extended after issues were raised regarding their behaviour. Dara, Anna and Wendy noted advancements for the perpetrator during or after the institution had been made aware of their behaviour:

*“… as bad as the bullying was, the complaint process and the investigation and the complaint against me was the worst part, by a mile… You always get difficult people in the workplace, but having to lose your sense of self and your reputation and what you stand for or potentially your job was the biggest source of stress for me and something that HR didn’t really recognize at all… They sold it just a bit of paperwork and no big deal to have to go through a process like that… But it’s the impact of the policy and the procedures. And what it does to people… there’s no cognizance of or effort to repair at all”.*

Dara’s comment was particularly poignant: “*I’d recommend people just leave, leave, leave, immediately. That would be like, just get out. There’s no way your university will look after you, just leave. Yeah*”.

### 4.4. Unravelled and Undone

There is a strong sense in the data that participants were ‘undone’ by the experience of being bullied in their HEI. In nine of the ten interviews, there had either been an attempt at resolution through the institution by the participants [albeit one that they felt was inadequate] or they had withdrawn from the position. Participants were changed by the experience either out of respect for their beliefs about themselves, higher education, or their health. As Breda put it: “*I don’t think I’ll ever fully be the same person. I’m always gonna be on my guard…*”.

In terms of health and well-being, there was a strong sense of lasting damage in the interview data. Many of the participants were upset when recounting their experiences, even in situations where the event was several years previously. Some anticipated being upset at the start of the interview, while others became upset as they recalled events. It was also evident that, having emerged from the situation, a sense of personal strength could be claimed from the experience. However, this fortitude came with a significant cost. Wendy made it very clear that although she survived [her word] bullying, it took a serious toll, and she knows she could not go through it again. Using a particularly powerful metaphor, she states:

*“Once, I survived though it was incredibly difficult… [it] broke me, and I don’t mean that I was broken, and that I fell apart. [UPSET] I didn’t. I stayed together, but I don’t have the resilience to withstand it a second time. And I know that I had a core that was cracked down the middle, and that’s the way I feel. So, the pot stays together. It’s held together. You don’t look like you are broken, but you know you are, and I know I could never go through it again, so I would leave rather than ever with stand that again, and I’m absolutely clear about that this is not something I could go through a second time”.*

A sense of organisational betrayal pervaded. A loss of trust was evident throughout the data. Participants were cynical and attributed this to their experience of having had faith in an organisational process that revealed itself to be unjust, unfair and, employing one participant’s word, dishonest. Cynicism was reflected in a shift in priorities and a greater focus on self-preservation, even at the expense of the workload of colleagues. There were references to disengagement, increased caution around others, and a decreased expectation of justice or fairness within the organisation, or even the sector. This is illustrated in Anna’s comments:

*“… loyalty, absolutely plummeted. Trust very much broke and I think especially at the start of the process, I felt like things like the anti-bullying policy had been developed in good faith and that they were there to protect me as an employee or other people like me. And I definitely came out the other side of it saying ‘the institution is covering its ass against lawsuits and it doesn’t care about well-being’… I also feel kind of a loss of faith in the institution of academia. Where people would say ‘Well, what if you went to [another HEI]’ But, why would I even bother?… it would just be the same thing again, but with new people who are terrible and new in unknown ways that I have to discover”.*

Wellness/wellbeing initiatives were particularly criticised. These were criticised for putting the responsibility back onto the target as well as proffering, what interviewees viewed as, overly simplistic and rather inane “fixes” for such complex negative interpersonal interactions. As both Breda and Anna argue, respectively: *“I also got told you know, oh, you know, go and do yoga. That washes away the stress of this place… So go away and do your Tai Chi. We provide Tai Chi, yoga at lunchtime. But when you come back from lunch, the problem is still waiting for you. You know what I mean?” “* and *“… like for a while actually every time I got an email from the director of HR being like it’s well-being week, go to free yoga like I just remember them in my grievance hearing and be like, fuck you [HR Director’s name]. I don’t wanna keep dwelling on this. But it’s hard not to when it feels like nothing was really done”.*

## 5. Discussion

The aim of this study was to explore how bullying and the response to it is experienced by university staff and how institutional or contextual factors impinge on that experience. To this end, we recruited and interviewed 10 people who were or who had recently worked in the Irish HE sector. It was not a requirement that participants were targets of bullying themselves, although most respondents had direct experience of being bullied, and several also of witnessing bullying. The data captured a wide diversity of experiences across different institutions, different career stages and different roles. Conceptually, the results affirm D’Cruz and Norohana’s multi-faceted conceptualisation of workplace bullying as both interpersonal, work-related and depersonalised, and including real and cyber forms, but also displaying the dynamics of relationality, institutionalisation and unethicality [29].

The themes emerging from the analysis converge in one central organizing construct; the traumatic nature of bullying, consistent with trauma defined as ‘exposure to an incident or series of events that are emotionally disturbing or life-threatening with lasting adverse effects on the individual’s functioning and mental, physical, social, emotional, and/or spiritual well-being’ [64] (p. 1). Bullying was experienced as traumatizing and processual, characterised as an undermining and damaging power-play between the target, the perpetrator and the institution. Participants recounted highly stressful experiences. Some were visibly upset while recalling events, even those that took place several years prior to the interview. Two participants recounted suicidal thoughts during the experience. While bullying experiences differed in nature and duration, there was a consistency to the emotions and reactions experienced by participants. Across the data, emotions included anger, bitterness, sorrow, frustration, deflation and guilt, and the strength of emotions was palpable. Yet it is worth noting that, despite this, all were keen to recount their story in detail, and several expressed the wish that doing so might contribute to improved responses to bullying in the sector. Features of the HEI working environment are exposed in these data that contribute to the traumatizing and [re]traumatizing nature of the experience, and to an understanding of the higher prevalence of bullying in the sector.

The participants in this study were deeply traumatized by bullying. The identification of the link between workplace bullying and trauma is not new; previous studies have demonstrated as association between workplace bullying and PTSD symptoms (e.g., [14,65,66,67]). Bullying experiences that are perceived as unexpected, uncontrollable or unjustified, generate fear or dread and worsen over time are deemed traumatic [68,69], all of which are present in these narratives. The personal suffering and the struggle to sense-make in the context of an institution dedicated to a ‘noble purpose’ were unmistakable. Bullying was unanticipated, perceived as undeserved, deliberate, intentional and sustained over unacceptable time periods. Participants experienced organisational unaccountability and a sense of betrayal. Their personal testimonies are consistent with Ferris’s reflection, based on 20 years of working with targets, of how the experiences of workplace bullying ‘*injure the soul and psyche of people*’, leading to severe functional impairment [70] (p. 3).

Metaphors, a shorthand often used to portray experiences that are difficult to describe, are powerful signifiers of the hurt experienced in bullying [71], and were evident in the narratives here, including being crushed, in a warzone, being thrown under a bus and being cracked at the core. The data also show how other aspects of the bullying experience can be traumatic, in particular the constant and sustained nature of it, such that every day is a difficult day. As the bullying experience progresses, stress results not only from actual incidents, but also from their anticipation, creating a torturesome working environment for the target (for example, Wendy felt physically unable to eat while on campus).

Previous explorations of how bullying is traumatising tend to focus on characteristics or vulnerabilities within the target, for example, the perturbation of conceptual systems and assumptions about the world [14], or exposure to previous life trauma [69]. The analysis here highlights the contribution of broader contextual factors, drawing on and affirming the attributes of relationality, institutionalisation and unethicality as underlying features of bullying [29], underscoring the significance of context.

Relationality refers to how bullying takes place within a relational context. The willingness of line managers or supervisors to abuse as opposed to benignly exercise power in the context of a hierarchical relationship was unexpected and, as a result, traumatising. Social and emotional power abuse took place against a backdrop of complex work relationships. Relationships were casualties for many participants, leading to violated respect and feelings of isolation. Both Anna and Wendy were aware that their working relationships with a senior academic were beyond repair, yet they still worked in the same physical and virtual environment. As Anna said: “*I mean, because even though this person isn’t the (line manager) anymore, they’re still in my department. They’re still a colleague. I still see them at meetings. They’re still research aligned to me like. I can’t really get away from them*”. The small number of Universities in Ireland limits mobility, highlighting the relevance of the national context. Holly’s complex experience led to the breakdown of several relationships, and could be regarded as an assault on relational dignity [29]. In Eva’s case, the relational context of her bullying experience blinded her to the bullying that was taking place, and ultimately led to a total breakdown of the relationship.

Institutionalization refers to the institution itself being ‘constitutive’ of abuse [72] thus normalising and embedding the problem within the organisation [29]. Organisational and social factors, germane to the institution, directly and indirectly facilitate, or even encourage, bullying and allow bullying to be ‘blind-eyed’, ultimately becoming systemic [3]. Intersecting with relationality, institutionalization was evident in the data here, signposting a number of contextual factors particular to HEIs and contributing to the traumatizing effects seen in these participants.

Power abuse, central to bullying in any context [29,56,73], is particularly complex in HEIs. Power relations in HEIs are unstable, exercised and resisted across multiple fora simultaneously, providing multiple opportunities for bullying. Certain academic relationships, for example, between research students and supervisors but also between junior and senior faculty, have traditional inbuilt and enduring power inequities [40]. Power relations can be exacerbated by precarity. In the current HE climate, perpetrators can use precarious workers to ‘build empires’, as noted by Dara. However, power inequities in HEIs do not just reflect traditional vertical power structures but also horizontal power differentials where the bully may be a colleague [74], as evidenced here in Ita’s case. As noted in the wider literature (e.g., [20,24]), gender inequity intersects already complex power relations and was perceived to play a role in the bullying experienced by Anna, Fran and Ita, all of whom encountered a management unwilling to challenge the power-base of long-established male academics. There is a sense in the data that controlling, aggressive or manipulative behaviour on the part of senior academics was rarely challenged. Their power appears to be inviolable, perhaps a function of the unique nexus of reward, positional, informational, and referent power in the HE context. This could be seen in Anna’s case, where her formal grievance was not upheld although many ‘concessions’ were granted; these were tantamount to an admission that the perpetrator was at fault, yet was also untouchable.

The study also highlights the significance of a unique feature of the HEI environment, namely, the transition from a student–supervisor relationship to a situation whereby the student becomes a staff member and the former supervisor their line manager. There are subtle differences between these two relationships, which, as seen in Eva’s case, can be exploited. Anna felt compromised by the fact that the perpetrator in her case would be most likely to be on any promotion panel, and therefore promotion was unlikely to ever be an option for her. The valorising of ‘expertise’, central to the HE environment, meant that a critical view of the self, presented to a target by a perpetrator who is a senior academic can be internalised, led to self-doubt or self-blame, also experienced by Eva and Dara.

Academic institutions are characterised by autonomy, individualism and discipline-related expertise, features that facilitate covert workplace bullying [75,76], rendering it difficult to recognise. Slow recognition was evident in these interviews, particularly for Eva who suffered many years of bullying, consistent with Merilalnen et al.’s description of bullying amongst academics as ‘sophisticated, psychologically emphasized, inappropriate behaviour which is difficult to label as bullying’ [77] (p. 160). Holly and Ita both noted the low level of accountability in HE, which contributed to organisational betrayal, resonant with the notions of non-centralised accountability and governance in professional organisations [21].

The data here echo pervasive discourses of neoliberal ideology, but reveal a hitherto under-studied aspect of the corporatization of universities; the traumatising impact of self-doubt with respect to professional identify. The valorisation of individuality and performativity inherent in the neoliberal university makes self-promotion a critical skill for advancement, a skill that can only be compromised by self-doubt and questioning. In this way, targets experience a type of double jeopardy, damage to their self-confidence and a weakened ability to engage with the systems that are designed for their advancement, which, at least for some, might constitute a release from the toxic relationship. The neoliberal agenda has also amplified precarity [78], straining power relations and leaving junior and untenured staff at risk of bullying, a situation that was noted by both Eva and Dara. Dara pointed out the risk of taking sick leave when bullied if in precarious employment: “*Gets to the point where you’re on half pay, then you’re not getting any pay and then your mortgage is due and all of these things. So… when you’re precarious, that’s days rather than months*”.

Finally, unethicality refers to the way in which workplace bullying goes against universal social rules of acceptability, and is a direct afront to ethics [29], citing [79]. Not only are the behaviours of humiliation, aggression, rumour spreading and gaslighting identified here counter to acceptable behaviour in organisations, but the failure of the organisation to address bullying seen in all 10 accounts speaks to a deeply distressing level of unethicality. In particular, the time delays experienced by targets, the default to mediation which, as Anna put it, was ‘ridiculous’, and signposting targets to tai chi and yoga in the face of abuse and exploitation are arguably morally offensive. The frequent references to known grievances raised against perpetrators in the past evidenced not only that bullying had become institutionalised, but that could be described as also an ‘afront to ethics’.

All three aspects of the framework combine in the accounts here of students being brought into bullying processes. Both the relationality and institutionalisation of bullying are evident when routine activities that manifest in daily relationships encourage bullying. The assessment and supervision of students could be so described. Particularly traumatising to targets in this study was the weaponizing of these processes, articulated by both Anna and Fran, with doubt being cast over their supervisory skills, expertise or approach to assessment. This highlights an unethical dimension to the power plays in bullying in HEIs. Academic staff have a moral and ethical responsibility, indeed a duty and care to students. Yet, the cases outlined in the data here expose the use of students in bullying tactics and the potential negative impact on students, placing the target in unacceptably compromised positions, trying to defend students while also defending themselves.

## 6. Conclusions

Notwithstanding the limitations of this study with respect to the sample size and the non-representative purposeful sampling methodology employed, this qualitative study of 10 persons who self-identified as directly or indirectly experiencing bullying highlights the traumatic nature of bullying in the HE sector. The findings are consistent with other qualitative studies regarding the negative impact on health and the poor response from the institution (e.g., [5,46,57,80]); however, the deeply traumatizing aspect is foregrounded in the narratives here, prompting a clarion call to the sector. Despite all the institutions having anti-bullying policies (all Irish universities, at the time of data collection, had anti-bullying or dignity at work policies in place), it is abundantly clear that they are not affording protection to all staff. The systems, processes and procedures in place to address bullying and provide redress are not nuanced enough to accommodate the complex behaviours and power plays involved in bullying in HEIs. They appear to assume a rationality behind behaviours, stripped of power dynamics, which the data here show is unrealistic, and which risks aggravating the damage already inflicted by bullying. Indeed, these findings suggest that the institutional response, or lack thereof, can sometimes be more [re]traumatising than the act itself, as it calls into question one’s worldview. Policies appear to be blinded to the fact that bullying is a deliberate power play and assume that parties act in good faith. None of the situations portrayed in these interviews could be so described.

While further studies, at both national and international level, would be required to consolidate the findings of this study and enhance the generalizability of the conclusions, it may be argued, nonetheless, that it is incumbent on HEIs to take a proactive and preventative approach, drawing on the literature produced within its own sector that highlights the complex and shifting relationships, the multilayered nature of power, the individualistic and competitive culture and the pressure to be market responsive. Individual cases of bullying are often reflecting a wider organisational culture that tolerates incivility and victimisation; therefore, institutional responses may be complex and will need to tackle the entire HEI ecosystem, requiring a more nuanced understanding of the power dynamics and organisational context. The relationship between the organisational context and higher levels of workplace bullying is well documented (e.g., [81,82]), and anti-bullying recommendations often include reducing relationship ambiguity (e.g., PhD supervisor and student), increasing autonomy and decision latitude, changing leadership behaviour, and systematic leadership development [83,84]. Such recommendations also pertain to the HEI sector, a sector that in 1995, Middlehurst [85] called upon to reevaluate traditional boundaries and internal demarcations between academic and non-academic, student and faculty, and part and full-time employees. Our findings suggest this call has yet to be answered.

## Data Availability

Data are unavailable due to privacy and ethical restrictions.
